# Coding-Complete Sequence of a SARS-CoV-2 Strain from an Omicron (B.1.1.529+BA.1) Variant Detected in Morocco

**DOI:** 10.1128/mra.00063-22

**Published:** 2022-04-14

**Authors:** Taha Chouati, Mouhssine Hemlali, Marouane Melloul, Sanaa Alaoui-Amine, Safae Rhoulam, Hamza Ghamaz, Maha Ouarab, Omar Askander, Lamiae Belayachi, Nadia Touil, Bouchra El Mchichi, Hicham Elannaz, Abdelillah Laraqui, Mostafa Elouennass, Khalid Ennibi, Elmostafa El Fahime

**Affiliations:** a Molecular Biology and Functional Genomics Platform, National Center for Scientific and Technical Research (CNRST), Rabat, Morocco; b Neuroscience and Neurogenetics Research Team, Faculty of Medicine and Pharmacy, University Mohammed V, Rabat, Morocco; c Center of Virology, Infectious and Tropical Disease, Mohamed V Military Teaching Hospital, Rabat, Morocco; d Microbiology Laboratory, Faculty of Medicine and Pharmacy, Mohammed V University, Mohamed V Military Teaching Hospital, Rabat, Morocco; e Microbiology and Molecular Biology Team, Center of Plant and Microbial Biotechnology, Biodiversity and Environment, Faculty of Sciences, Mohammed V University of Rabat, Rabat, Morocco; f Research Genetics Center of the Cheikh Zaid Foundation, Abulcasis International University of Health Sciences, Rabat, Morocco; g Research Genetics Center of the Cheikh Zaid Foundation, Rabat, Morocco; DOE Joint Genome Institute

## Abstract

Here, we describe the coding-complete sequence of severe acute respiratory syndrome coronavirus 2 (SARS-CoV-2) strain HM36, identified as a strain of concern of B.1.1.529+BA (Omicron).

## ANNOUNCEMENT

The global pandemic affecting the world since the start of 2020 is caused by severe acute respiratory syndrome coronavirus 2 (SARS-CoV-2) (*Betacoronavirus* genus, *Betacoronaviridae* family) (1). The monitoring of the spread of the variants of concern (VOCs) is of crucial importance (2).

In late November 2021, The World Health Organization classified the B.1.1.529 variant (Omicron) as a VOC which shows a high number of mutations at the level of the protein S gene and seems to spread at a higher rate than the previously dominant variants (3). In Morocco, the first case of Omicron was reported on 15 December 2021, and it quickly spread to account for about 60% of the sequenced samples in the first week of January 2022.

Here, we describe the coding-complete sequence of SARS-CoV-2 sample HM36, which was collected from an asymptomatic female patient at Ibn Rochd Hospital of Casablanca, Morocco, and tested positive for SARS-CoV-2 by quantitative reverse transcription (RT)-PCR using a GeneProof COVID-19 Plus Real Amp kit.

The swab sample was received at the National Center for Scientific and Technical Research on 16 December 2021 as part of the ongoing SARS-CoV-2 variant surveillance. Viral extraction was performed using a MagPurix viral RNA extraction kit (Zinexts Life Science, China). Whole-genome sequencing was performed using Ion Proton technology (Applied Biosystems, USA). Briefly, cDNA was obtained through reverse transcription using a VILO cDNA synthesis kit (Invitrogen, Thermo Fisher Scientific, USA). A DNA library was prepared using the Ion AmpliSeq SARS-CoV-2 research panel (Thermo Fisher, USA) and then processed with an Ion Chef instrument for template preparation and chip loading. The sequencing was performed using an Ion S5 sequencer.

The obtained sequences (2,423,738 reads; mean length, 184 bp) were quality controlled and assembled using SPAdes v3.14.1 (4), and then a consensus sequence was generated using Unipro UGENE v38.1 (5), with the reference sequence being the Wuhan-Hu-1 sequence obtained from NCBI (GenBank accession no. NC_045512). Both tools were used with default parameters. The generated sequence was 29,804 bp long with a GC content of 37.96%.

**FIG 1:**
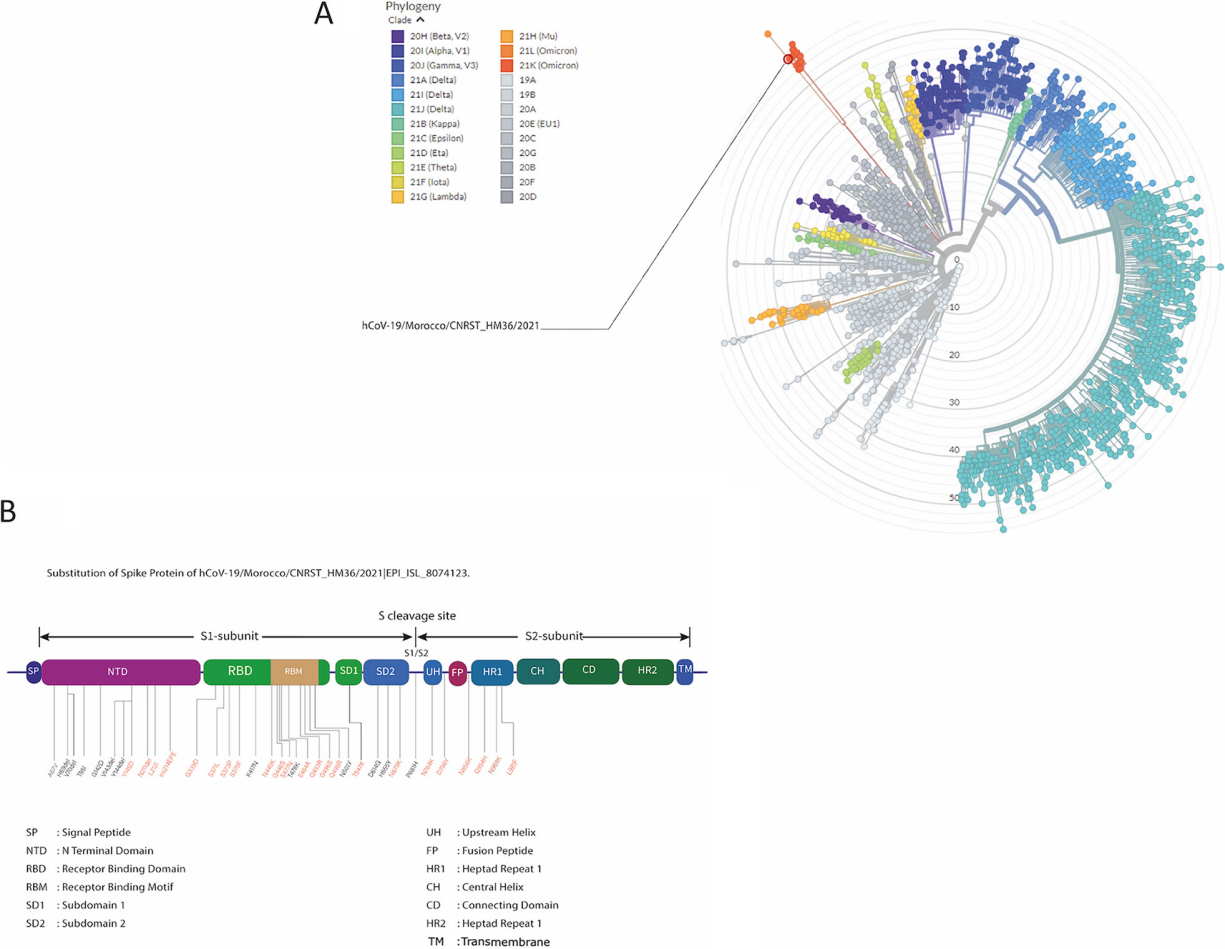
(A) Radial representation of the phylogenetic relatedness of SARS-CoV-2 genomes with all the named lineages and sublineages of lettered variants highlighted in different colors. SARS-CoV-2 CNRST_HM36/2021 l EPI_ISL_8074123 clearly belongs to the 21k lineage (Omicron) represented in dark orange. (B) Schematic representation of SARS-CoV-2 CNRST_HM36/2021 l EPI_ISL_8074123 spike protein showing amino acid changes and their positions on different domains. The majority of changes concerned the NTD and RBD in the S1-subunit region, while the CH, CD, HR2, and TM domains in the S2-subunit remained unchanged. Amino acid changes characterizing the Omicron variant are shown in red, while the ones in black are found among other variants.

Lineage identification using Pangolin (https://pangolin.cog-uk.io/) showed that the strain belonged to the B.1.1.529+BA lineage (Omicron), first detected in South Africa (Fig. 1A). CoV-GLUE analysis (http://cov-glue.cvr.gla.ac.uk/) revealed that the spike protein contained 36 modifications, including 29 amino acid (aa) substitutions, 6 deletions, and 1 insertion. A total of 15 genes showed no aa changes compared to the Wuhan virus (Table 1).

**TABLE 1:** Amino acids changes of strain hCoV-19/Morocco/CNRST_HM36/2021|EPI_ISL_8074123 compared with the Wuhan-Hu-1 SARS-CoV-2 reference sequence (GenBank accession number NC_045512)

Protein	Amino acid change(s)
NSP1	No aa changes
NSP2	No aa changes
NSP3	K38R, S1265del, L1266I, A1892T
NSP4	T14I, T492I
NSP5	P132H
NSP6	L105del, S106del, G107del, I189V
NSP7	No aa changes
NSP8	No aa changes
NSP9	No aa changes
NSP10	No aa changes
NSP11	No aa changes
NSP12	P323L
NSP13	No aa changes
NSP14	I42V
NSP15	No aa changes
NSP16	No aa changes
Spike	A67V, H69del, V70del, T95I, G142D, V143del, Y144del, Y145del, N211del, L212I, ins214EPE, G339D, S371L, S373P, S375F, K417N, N440K, G446S, S477N, T478K, E484A, Q493R,G496S, Q498R, N501Y, T547K, D614G, H655Y, N679K, P681H, N764K, D796Y, N856K, Q954H, N969K, L981F
NS3	No aa changes
E	T9I
M	D3G, Q19E, A63T
NS6	No aa changes
NS7a	No aa changes
NS7b	No aa changes
NS8	No aa changes
N	P13L, E31del, R32del, S33del, R203K, G204R

Among the observed amino acid changes in sample HM36 (Table 1), there is a marked overlap with other VOCs (6), namely, the widespread N501Y and D614G, but also K417N, found in the Beta and Gamma variants, T478K in the Delta variant, and P681H in the Alpha variant. E484A is also noticeable, as a similar change is found in Alpha, Beta, and Gamma variants, albeit to a different amino acid (E484K) (7). However, other changes seem to be specific to the Omicron variant, such as G446S, G496S, T547K, N856K, and L981F (Fig. 1B).

### Data availability.

The SARS-CoV-2 genome sequence was submitted to the GISAID database under the identifier EPI_ISL_8074123 (https://www.epicov.org/epi3/frontend#6111c3 after login process) and to NCBI GenBank under the accession number OM011974. The raw data are available under BioProject number PRJNA817433.
